# Patients’ experiences of GP consultations following the introduction of the new GP contract in Scotland: a cross-sectional survey

**DOI:** 10.3399/BJGP.2023.0239

**Published:** 2024-01-23

**Authors:** Kieran D Sweeney, Eddie Donaghy, David Henderson, Huayi Huang, Harry HX Wang, Andrew Thompson, Bruce Guthrie, Stewart W Mercer

**Affiliations:** Usher Institute, College of Medicine and Veterinary Medicine, University of Edinburgh, Edinburgh, UK.; Usher Institute, College of Medicine and Veterinary Medicine, University of Edinburgh, Edinburgh, UK.; Usher Institute, College of Medicine and Veterinary Medicine, University of Edinburgh, Edinburgh, UK.; Usher Institute, College of Medicine and Veterinary Medicine, University of Edinburgh, Edinburgh, UK.; School of Public Health, Sun Yat-Sen University, Guangzhou, China.; School of Social and Political Science, University of Edinburgh, Edinburgh, UK.; Usher Institute, College of Medicine and Veterinary Medicine, University of Edinburgh, Edinburgh, UK.; Usher Institute, College of Medicine and Veterinary Medicine, University of Edinburgh, Edinburgh, UK.

**Keywords:** general practice, health inequalities, inverse care law, patient satisfaction, remote and rural, remote consultation, Scottish GP contract

## Abstract

**Background:**

The new Scottish GP contract commenced in April 2018 with a stated aim of mitigating health inequalities.

**Aim:**

To determine the health characteristics and experiences of patients consulting GPs in deprived urban (DU), affluent urban (AU), and remote and rural (RR) areas of Scotland.

**Design and setting:**

In 2022, a postal survey of a random sample of adult patients from 12 practices who had consulted a GP within the previous 30 days was undertaken.

**Method:**

Patient characteristics and consultation experiences in the three areas (DU, AU, RR) were evaluated using validated measures including the Consultation and Relational Empathy (CARE) Measure and Patient Enablement Instrument (PEI).

**Results:**

In total, 1053 responses were received. In DU areas, multimorbidity was more common (78% versus 58% AU versus 68% RR, *P*<0.01), complex presentations (where the consultation addressed both psychosocial and physical problems) were more likely (16% versus 10% AU versus 11% RR, *P*<0.05), and more consultations were conducted by telephone (42% versus 31% AU versus 31% RR, *P*<0.01). Patients in DU areas reported lower satisfaction (82% DU completely, very, or fairly satisfied versus 90% AU versus 86% RR, *P*<0.01), lower perceived GP empathy (mean CARE score 38.9 versus 42.1 AU versus 40.1 RR, *P*<0.05), lower enablement (mean PEI score 2.6 versus 3.2 AU versus 2.8 RR, *P*<0.01), and less symptom improvement (*P*<0.01) than those in AU or RR areas. Face-to-face consultations were associated with significantly higher satisfaction, enablement, and perceived GP empathy than telephone consultations in RR areas (all *P*<0.05).

**Conclusion:**

Four years after the start of the new GP contract in Scotland, patients’ experiences of GP consultations suggest that the inverse care law persists.

## Introduction

Population ageing and the increasing prevalence of multimorbidity pose significant challenges to healthcare services globally. In Scotland, as in the rest of the UK, workforce shortages and the enduring impact of the COVID-19 pandemic have added to these pressures.^1^^,^^2^ Meanwhile, health inequalities in Scotland are widening, with disparities not only across socioeconomic groups, but also by gender, ethnicity, and geography.^3^ The latter is of particular relevance in Scotland, where over 15% of the population live in remote and rural areas.^3^^,^^4^

Health inequalities are compounded by the inverse care law — first described in the NHS over 50 years ago — that states: *‘the availability of good medical care tends to vary inversely with the need for it in the population served’*.^5^^–^9 Previous research has shown how the inverse care law operates in primary care in deprived areas of Scotland, where GP consultations are shorter, less patient centred, have lower perceived GP empathy, lower enablement for patients with complex needs, and poorer outcomes compared with affluent areas.^6^^–^10

Policies of reform in primary care have been central to the efforts of healthcare systems globally in addressing the challenges of population ageing, multimorbidity, and health inequalities.^11^^,^^12^ A recent scoping review found considerable heterogeneity in how reform plays out in different systems and noted that the views of patients are often overlooked in their design and evaluation.^12^

**Table:** How this fits in

The mitigation of health inequalities was a stated aim of the new GP contract introduced in Scotland in April 2018. This survey of patients from deprived urban, affluent urban, and remote and rural areas of Scotland found that those in deprived urban areas had the greatest health needs, with higher levels of multimorbidity, complex presentations, coexisting mental–physical multimorbidity, and frequency of GP attendance. The same group also had the poorest experience of GP consultations, with lower levels of satisfaction, perceived GP empathy, enablement, and symptom improvement. Solutions are required to reverse the long-standing inverse care law that based on these data would appear not to have been improved by the new GP contract in Scotland.

In Scotland, health and social care policy is a devolved responsibility and healthcare delivery is organised within 14 regional health boards. In 2016, the Scottish Government abolished the Quality and Outcomes Framework and reorganised GP practices into geographic clusters. In April 2018 a new GP contract further redesigned primary care services in Scotland,^13^ with one stated aim of reducing health inequalities. Key changes included an expansion of the primary care multidisciplinary team (MDT), designed to allow GPs to focus more time on patients with complex multimorbidity.^13^ Previous work in deprived areas has shown that longer consultations lead to higher enablement of patients with complex needs,^14^ and, when combined with a patient-centred empathic approach, improvements in quality of life and wellbeing.^15^

The impact of the 2018 GP contract on health inequalities in Scotland — either socioeconomic or geographic — is unknown. The authors’ recent qualitative research found that GPs in rural areas view the contract as too ‘city centric’ and some in deprived areas feel it has failed to free up GP time to spend with patients with complex needs.^16^^,^^17^

The aim of this study was to survey the health needs and experiences of patients consulting GPs in three population settings: deprived urban (DU), affluent urban (AU), and remote and rural (RR) areas of Scotland.

## Method

### Study design

This study used a postal questionnaire of patients who had recently consulted a GP in a purposive sample of 12 practices across three health boards in Scotland.

### Sampling, recruitment, and data collection

Three health boards were selected to give a range of geographic and socioeconomic characteristics. Four clusters were recruited from each of these health boards, and one practice recruited from each cluster. A random sample of 6291 adult patients (aged ≥18 years) who had consulted a GP within the past 30 days were identified from practice records. The sample size in each practice ranged from 159 to 748. This depended on the size of the practice list and was weighted towards areas of high deprivation where lower response rates were anticipated. The sample size was chosen based on the authors’ previous work on GP consultations, which found significant differences between affluent and deprived populations,^7^^,^^8^ and on the time and resource constraints of the study.

Questionnaires were sent with a cover letter and participant information sheet, and returned using stamped addressed envelopes. As a result of funding and time constraints, no reminders were sent. Sampling of patients took place in the week commencing 22 August 2022, and questionnaires were posted between 31 August and 15 September 2022. Collection of responses ran until 30 November 2022.

Out of 6291 patients from 12 practices, 1053 responded (response rate 17%). Response rates were higher in AU areas (27%, *n* = 273/1000) than in RR areas (20%, *n* = 332/1680) and DU areas (12%, *n* = 448/3611). The distribution of responders’ ages differed significantly from those of non-responders, with older patients relatively overrepresented among responders. There were no differences in gender profiles between responders and non-responders. Deprivation scores did not differ between responders and non-responders in the AU or RR groups, but in the DU areas the responders were significantly less deprived than non-responders (Supplementary Tables S1 and S2).

### Instruments used

The content of the questionnaire was based on the original study conducted by the senior author in 2007.^6^ Sociodemographic information included responders’ age, gender, employment status, living arrangement, and ethnicity. Deprivation status was obtained from the Scottish Index of Multiple Deprivation (SIMD) linked with each patient’s postcode, and recorded in deciles, with one being the most deprived and 10 the least deprived.^18^ Health characteristics included self-rated general health over the past 12 months; frequency of GP attendance over the past 12 months; and current disability or long-term illness.^6^ Depression and anxiety symptoms were measured using the Patient Health Questionniare-4 (PHQ-4).^19^

Multimorbidity was assessed using a checklist of 17 common chronic conditions, with space to add additional conditions not listed, as in the authors’ previous studies.^6^^,^^7^

Characteristics of the GP encounter included the consultation type (face-to-face, telephone, video, or home visit); time elapsed in weeks since the last consultation; and the number and type of problems discussed (physical, emotional or psychological, social, administrative, or other).^6^

Patients’ perception of GP empathy was assessed using the Consultation and Relational Empathy (CARE) measure.^20^ This was assessed alongside overall satisfaction with the consultation and the likelihood of recommending the doctor to family and friends.^6^ Consultation outcomes included the six-item Patient Enablement Instrument (PEI), measuring the impact of the encounter on a patient’s ability to cope with and understand their health problems.^21^ Perceived improvement in symptoms since the consultation was also assessed.^22^

### Data analysis

Descriptive analysis was performed using SPSS version 27. Practices were grouped according to whether they served mainly DU, AU, or RR areas. Differences between the three population groups were assessed using the appropriate parametric or non-parametric tests (Kruskal–Wallis or ANOVA) with further pairwise comparisons conducted (using Mann–Whitney tests or independent *t*-tests) where a significant difference was found on three-way testing.

Consultation experiences were compared between face-to-face consultations (FTFC) and telephone consultations (TC). As a result of the low numbers, home visits (*n* = 11) and video consultations (*n* = 1) were excluded from these comparisons. When analysing the types of problems discussed in the consultation, ‘complex problems’ were defined as a combination of a physical problem(s) plus a psychological and/or social problem(s).^6^

## Results

The sociodemographic characteristics of the patients who took part in the survey are shown in Table 1. In total, 60% (*n* = 609/1053) of responders were female, which was similar across the three groups. Time elapsed since the consultation was also consistent between groups, ranging from ‘<1 week’ (9%, *n* = 92/1035) to ‘≥4 weeks’ (42%, *n* = 432/1035), with a median response of ‘2–3 weeks’ (32%, *n* = 332/1035). The DU group had a median deprivation decile score of three, compared with 10 for the AU group and five for the RR group (*P*<0.01). The DU group had the lowest mean age at 60.9 years, compared with 62.0 years in the AU group, and 65.8 years in the RR group (*P*<0.01). Rates of unemployment were highest in the DU group and rates of retirement were highest in the RR group (both *P*<0.01).

**Table 1.** Sociodemographic characteristics of participating patients in affluent urban, deprived urban, and remote and rural areas^a^

**Characteristic**	**RR (*N* = 332)**	**AU (*N* = 273)**	**DU (*N* = 448)**	**Overall group comparison, *P*-value**	**Two-way group comparisons, *P*-value**		

					**DU versus AU**	**AU versus RR**	**DU versus RR**
**Gender, % (*n*)**	322	264	430	0.491	—	—	—
Male	39.8 (128)	42.8 (113)	37.9 (163)				
Female	60.2 (194)	56.4 (149)	61.9 (266)				
Other	0.0 (0)	0.8 (2)	0.2 (1)				

**Age, years, mean (SD)**	65.8 (14.5)	62.0 (17.6)	60.9 (14.9)	<0.001	0.001	<0.001	0.405

**Age group, years, % (*n*)**	324	268	436	<0.001	0.217	0.032	<0.001
<45	9.3 (30)	17.5 (47)	14.2 (62)				
45–64	31.5 (102)	29.5 (79)	40.8 (178)				
≥65	59.3 (192)	53.0 (142)	45.0 (196)				

**SIMD decile, % (*n*)**	332	273	447	<0.001	<0.001	<0.001	<0.001
1 (most deprived)	0.0 (0)	0.0 (0)	19.5 (87)				
2	0.3 (1)	0.0 (0)	23.5 (105)				
3	4.2 (14)	1.5 (4)	13.0 (58)				
4	32.2 (107)	5.5 (15)	12.1 (54)				
5	20.2 (67)	2.2 (6)	6.7 (30)				
6	22.6 (75)	0.4 (1)	8.1 (36)				
7	15.1 (50)	3.3 (9)	4.9 (22)				
8	2.1 (7)	6.2 (17)	2.9 (13)				
9	3.3 (11)	15.0 (41)	6.5 (29)				
10 (least deprived)	0 (0)	65.9 (180)	2.9 (13)				

**Job status, % (*n*)**	325	267	437	0.007	0.104	0.321	0.002
Employed	34.5 (112)	40.1 (107)	39.8 (174)				
Retired	58.8 (191)	52.1 (139)	41.2 (180)				
Unemployed, looking	0.3 (1)	0.7 (2)	2.1 (9)				
Unemployed, unable	5.2 (17)	1.9 (5)	14.4 (63)				
In education	0.3 (1)	2.2 (6)	0.5 (2)				
Other	0.9 (3)	3.0 (8)	2.1 (9)				

**Living arrangement, % (*n*)**	325	267	435	0.120	—	—	—
Alone	24.6 (80)	24.0 (64)	35.2 (153)				
With partner	68.6 (223)	69.7 (186)	53.3 (232)				
With someone else	6.8 (22)	6.4 (17)	11.5 (50)				

**Time elapsed since consultation,^b^ % (*n*)**	329	269	437	0.803	—	—	—
<1 week	9.1 (30)	8.9 (24)	8.7 (38)				
1–2 weeks	18.2 (60)	17.1 (46)	16.7 (73)				
2–3 weeks	28.3 (93)	34.6 (93)	33.4 (146)				
≥4 weeks	44.4 (146)	39.4 (106)	41.2 (180)				

a *Overall group comparisons used Kruskal–Wallis tests, except for Age and SIMD where ANOVA was used. Two-way comparisons used Mann–Whitney tests, except for Age and SIMD, which used independent* t*-tests. Two-way comparisons were not performed where the overall (three-way) group comparison was not significant.*

b *Categories for ‘Time elapsed since consultation’ are shown as in the original survey. AU = affluent urban. DU = deprived urban. RR = remote and rural. SD = standard deviation. SIMD = Scottish Index of Multiple Deprivation.*

Table 2 summarises the health characteristics of patients in the three groups. The DU group had significantly worse general health, higher rates of disability or long-term illness, and higher PHQ-4 scores (depression and anxiety) than both other groups (*P*<0.01). There was a significantly higher proportion of patients with multimorbidity (≥2 conditions) in the DU group than in both other groups (78% versus 58% AU versus 68% RR, *P*<0.01). Coexisting mental–physical multimorbidity was also highest in the DU group (36% versus 18% AU versus 19% RR, *P*<0.01). Compared with the AU group, patients in the RR group had significantly higher multimorbidity, poorer general health, and higher levels of disability or long-term illness (all *P*<0.01).

**Table 2.** Health characteristics of participating patients in affluent urban, deprived urban, and remote and rural areas^a^

**Characteristic**	**RR (*N* = 332)**	**AU (*N* = 273)**	**DU (*N* = 448)**	**Overall group comparison, *P*-value**	**Two-way group comparisons, *P*-value**		

					**DU versus AU**	**AU versus RR**	**DU versus RR**
**General health, *N* % (*n*)**	331	272	443	<0.001	<0.001	<0.001	<0.001
Very good	15.7 (52)	21.0 (57)	8.1 (36)				
Good	32.6 (108)	43.0 (117)	23.7 (105)				
Fair	37.8 (125)	26.8 (73)	39.3 (174)				
Bad	11.8 (39)	7.7 (21)	23.0 (102)				
Very bad	2.1 (7)	1.5 (4)	5.9 (26)				

**Multimorbidity, number of conditions, *N* % (*n*)**	332	273	444	<0.001	<0.001	<0.001	0.009
0	10.2 (34)	14.7 (40)	7.0 (31)				
1	22.3 (74)	27.1 (74)	15.5 (69)				
2	20.8 (69)	26.7 (73)	23.9 (106)				
≥3	46.7 (155)	31.5 (86)	53.6 (238)				

**Multimorbidity, number of conditions, mean (SD)**	2.6 (1.8)	2.1 (1.6)	3.0 (2.0)	<0.001	<0.001	<0.001	0.003
**Mental–physical multimorbidity,^b^ % (*n*)**	18.7 (62)	17.6 (48)	35.6 (158)	<0.001	<0.001	0.729	<0.001
**Disability or long-term illness,^b^ % (*n*)**	47.4 (153)	25.0 (68)	56.8 (251)	<0.001	<0.001	<0.001	0.010

**PHQ-4 score, *N* % (*n*)**	326	271	435	<0.001	<0.001	0.367	<0.001
Normal	72.4 (236)	69.0 (187)	48.5 (211)				
Mild (3–5)	16.6 (54)	18.1 (49)	20.5 (89)				
Moderate (6–8)	6.1 (20)	8.1 (22)	13.6 (59)				
Severe (9–12)	4.9 (16)	4.8 (13)	17.5 (76)				

a *Overall group comparisons used Kruskal–Wallis tests, except for general health where ANOVA was used. Two-way comparisons used Mann–Whitney tests, except for general health, which used independent* t*-tests.*

b *For ‘mental–physical multimorbidity’ and ‘disability or long-term illness’, N values differ due to missing data AU = Affluent urban. DU = deprived urban. PHQ-4 = Patient Health Questionniare-4. RR = remote and rural. SD = standard deviation.*

Patterns of consulting (Table 3) differed significantly across the three groups. Patients in the DU group were most likely to have had a TC and least likely to have a FTFC in the previous 4 weeks, compared with both other groups (both *P*<0.01). Frequency of attendance over the past 12 months was significantly higher in the DU and RR groups than in the AU group (*P*<0.01).

**Table 3.** Patterns of consulting of participating patients in affluent urban, deprived urban, and remote and rural areas^a^

**Characteristic**	**RR (*N* = 332)**	**AU (*N* = 273)**	**DU (*N* = 448)**	**Overall group comparison, *P*-value**	**Two-way group comparisons, *P*-value**		

					**DU versus AU**	**AU versus RR**	**DU versus RR**
**Attendances in past year, mean (SD)**	4.6 (3.6)	3.9 (4.0)	5.3 (4.3)	<0.001	<0.001	0.001	0.056

**Attendances in past year, grouped, % (*n*)**	319	271	428	<0.001	<0.001	<0.001	0.096
1–3	45.5 (145)	57.2 (155)	38.8 (166)				
4–6	34.5 (110)	32.8 (89)	38.8 (166)				
>6	20.1 (64)	10.0 (27)	22.4 (96)				

**Consultation type, % (*n*)**	328	269	438	<0.001	<0.001	0.542	0.002
Face to face	67.7 (222)	68.0 (183)	56.8 (249)				
Telephone	30.8 (101)	31.2 (84)	42.0 (184)				
Home visit	1.5 (5)	0.4 (1)	1.1 (5)				
Video	0.0 (0)	0.4 (1)	0.0 (0)				

**Number of problems, % (*n*)**	309	262	391	<0.001	<0.001	0.078	0.001
1	56.3 (174)	63.4 (166)	46.0 (180)				
2	34.0 (105)	29.4 (77)	36.1 (141)				
≥3	9.7 (30)	7.3 (19)	17.9 (70)				

**Type of problem(s), % (*n*)**	324	267	434	0.018	0.019	0.896	0.020
Physical	73.5 (238)	73.8 (197)	64.3 (279)				
Psychosocial	4.3 (14)	5.2 (14)	7.6 (33)				
Physical and psychosocial^b^	10.8 (35)	9.7 (26)	16.1 (70)				
Administrative or other	11.4 (37)	11.2 (30)	12.0 (52)				

a *Overall group comparisons used Kruskal–Wallis tests. Two-way comparisons used Mann–Whitney tests.*

b *Complex presentation.* *AU = affluent urban. DU = deprived urban. PHQ-4 = Patient Health Questionniare-4. RR = remote and rural. SD = standard deviation.*

Patients in the DU group were significantly more likely to present with ≥3 problems than those in both other groups (*P*<0.01). Discussion of complex problems in the consultation was also more common in the DU group compared with both other groups (16% versus 10% AU versus 11% RR, *P*<0.05), whereas presentations comprising solely physical problems were least common in this group (*P*<0.05). The number and nature of presenting problems, and the type of consultation, did not vary significantly between the AU and RR groups.

There were significant differences in patients’ consultation experiences between the three groups (Table 4). Patients in the DU group reported significantly lower satisfaction at consultation and less symptom improvement following the consultation than both other groups, as well as significantly lower perceived GP empathy than the AU group (all *P*<0.01). Patient enablement was significantly higher in the AU group than both other groups (*P*<0.01).

**Table 4.** Consultation experience of participating patients in affluent urban, deprived urban, and remote and rural areas^a^

**Characteristic**	**RR (*N* = 332)**	**AU (*N* = 273)**	**DU (*N* = 448)**	**Overall group comparison, *P*-value**	**Two-way group comparisons, P-value**		

					**DU versus AU**	**AU versus RR**	**DU versus RR**
**Satisfaction with consultation, % (*n*)**	329	271	443	0.004	0.003	0.696	0.009

Completely dissatisfied	2.1 (7)	2.6 (7)	5.2 (23)				
Very dissatisfied	1.8 (6)	1.8 (5)	2.5 (11)				
Fairly dissatisfied	4.0 (13)	1.5 (4)	4.7 (21)				
Neutral	6.1 (20)	4.4 (12)	5.9 (26)				
Fairly satisfied	16.4 (54)	16.2 (44)	19.2 (85)				
Very satisfied	27.4 (90)	32.5 (88)	28.4 (126)				
Completely satisfied	42.2 (139)	41.0 (111)	34.1 (151)				

**Likelihood to recommend doctor, % (*n*)**	329	268	440	0.276			
Definitely not	1.2 (4)	0.7 (2)	2.3 (10)				
Probably not	4.3 (14)	2.2 (6)	3.4 (15)				
Not sure	5.2 (17)	6.7 (18)	6.6 (29)				
Probably yes	23.4 (77)	23.9 (64)	26.1 (115)				
Definitely yes	66.0 (217)	66.4 (178)	61.6 (271)				

**PEI score, mean (SD)**	2.8 (3.4)	3.2 (3.2)	2.6 (3.1)	0.008	0.007	0.029	0.508

**CARE score, mean (SD)**	40.1 (10.5)	42.1 (8.1)	38.9 (11.1)	0.026	0.002	0.187	0.186

**Effect on symptoms, % (*n*)**	245	187	351	0.003	0.002	0.314	0.019
No improvement or worse	24.5 (60)	24.6 (46)	31.9 (112)				
Minor improvement	21.2 (52)	18.7 (35)	21.7 (76)				
Moderate improvement	32.7 (80)	27.3 (51)	30.8 (108)				
Major improvement	21.6 (53)	29.4 (55)	15.7 (55)				

a *Overall group comparisons used Kruskal–Wallis tests. Two-way comparisons used Mann–Whitney tests. Two-way comparisons were not performed where the overall (three-way) group comparison was not significant. AU = affluent urban. CARE = Care and Relational Empathy measure. DU = deprived urban. PEI = Patient Enablement Instrument. RR = remote and rural. SD = standard deviation.*

Overall, face-to-face consultations (Table 5) were associated with higher ratings of empathy, enablement, satisfaction, and recommendation likelihood than TC (all *P*<0.05). However, when analysed by group, only the RR group demonstrated a significant difference between TC and FTFC in terms of consultation experience.

**Table 5.** Comparing consultation experience in face-to-face and telephone consultation of participating patients in affluent urban, deprived urban, and remote and rural areas^a^

**Setting and characteristic**	**Face to face**	**Telephone**	***P*-value**
**All settings**			
Total patients (*N* = 1023), % (*n*)	63.9 (654)	36.1 (369)	
Satisfaction with consultation, mean (SD)	5.8 (1.5)	5.6 (1.4)	0.017
Likelihood to recommend doctor, mean (SD)	4.5 (0.8)	4.3 (1.0)	0.003
PEI score, mean (SD)	3.1 (3.3)	2.5 (3.0)	0.001
CARE score, mean (SD)	40.7 (9.9)	38.6 (11.0)	0.007
Effect on symptoms, mean (SD)	1.5 (1.1)	1.4 (1.1)	0.138

**RR**			
Total patients (*N* = 323), % (*n*)	68.7 (222)	31.3 (101)	
Satisfaction with consultation, mean (SD)	5.9 (1.4)	5.6 (1.5)	0.029
Likelihood to recommend doctor, mean (SD)	4.6 (0.8)	4.3 (1.0)	0.002
PEI score, mean (SD)	3.4 (3.7)	1.6 (2.3)	<0.001
CARE score, mean (SD)	41.5 (9.3)	36.5 (12.3)	0.002
Effect on symptoms, mean (SD)	1.6 (1.1)	1.4 (1.0)	0.111

**AU**			
Total patients (*N* = 267), % (*n*)	68.5 (183)	31.5 (84)	
Satisfaction with consultation, mean (SD)	5.9 (1.4)	6.0 (1.2)	0.931
Likelihood to recommend doctor, mean (SD)	4.6 (0.7)	4.4 (0.9)	0.157
PEI score, mean (SD)	3.2 (3.1)	3.3 (3.4)	0.741
CARE score, mean (SD)	42.2 (7.8)	41.8 (9.0)	0.927
Effect on symptoms, mean (SD)	1.6 (1.1)	1.8 (1.3)	0.192

**DU**			
Total patients (*N* = 433), % (*n*)	57.5 (249)	42.5 (184)	
Satisfaction with consultation, mean (SD)	5.6 (1.7)	5.5 (1.6)	0.179
Likelihood to recommend doctor, mean (SD)	4.4 (0.9)	4.4 (1.0)	0.639
PEI score, mean (SD)	2.7 (3.1)	2.5 (3.1)	0.443
CARE score, mean (SD)	39.0 (11.4)	38.6 (10.7)	0.491
Effect on symptoms, mean (SD)	1.4 (1.1)	1.3 (1.1)	0.326

a P*-values calculated using Mann–Whitney tests. This analysis excludes those patients who had home visits (*n *= 11) or video consultations (*n *= 1). The percentages reported are row percentages. AU = affluent urban. CARE = Care and Relational Empathy measure. DU = deprived urban. PEI = Patient Enablement Instrument. RR = remote and rural. SD = standard deviation.*

## Discussion

### Summary

This study found that patients in DU areas had the greatest health needs, with higher levels of multimorbidity, complex presentations, and frequency of GP attendance compared with the other two groups. The DU group also had the poorest experience of GP consultations, with lower satisfaction, perceived GP empathy, enablement, and symptom improvement compared with the AU or RR groups.

Although there were some demographic and health status differences between the AU and RR groups, there were no differences in the type of consultation, number or type of problems discussed, or most of the measures relating to consultation experience (with the exception of the PEI, which was lower in the RR group).

In the RR group, FTFC were associated with better consultation experience than telephone consultations. The fact that this difference was not evident in either the DU or AU groups suggests that the significant differences in consultation experience between these groups is not attributable to the higher proportion of TC in the DU group.

### Strengths and limitations

The strengths of this study were its relatively large sample size, the use of a bespoke questionnaire including several validated measures, and the inclusion of three populations of interest that have not been directly compared before. The main limitation was the relatively low response rate of 17% (12% DU versus 27% AU versus 20% RR). This is not dissimilar to that seen in the Scottish Government’s bi-annual national patient surveys, which obtains overall response rates of 20–25%, with much lower rates in deprived areas.^23^ The DU group was the biggest group in the current survey (3611 of 6291 patients), so the low response rate in this group reduced the overall figure considerably. The original plan had been to collect questionnaires within GP practices immediately after the consultation (where 70% response rates have been obtained),^6^ but this was not possible because of the COVID-19 pandemic, when most FTFC in general practice stopped. Additionally, postal follow-ups were not possible because of funding constraints.

The comparison groups in this study were based on area-based deprivation scores derived from patients’ postcodes (SIMD),^18^ rather than the individual deprivation level. The use of individual measures of socioeconomic position (such as employment and education level) may have resulted in different findings. However, SIMD scores are widely used in Scotland by researchers, health boards, and the Scottish Government.

In all groups, responders differed from non-responders in terms of age, and, in the DU group, responders were significantly less deprived than non-responders (Supplementary Table S1). Although this has implications for the generalisability of the findings, the fact that responders were the ‘least deprived of the deprived’ suggests that the significant differences found between the DU and the other two groups may in fact be an underestimate, as has been shown elsewhere.^24^

Although the current study repeated many measures used in the authors’ 2007 and 2016 studies,^6^^,^^7^ direct comparison is difficult because of the different timeframes in which responses were gathered. In the 2007 study, responses were gathered immediately after the consultation.^6^ In the present study, 42% of questionnaires were completed ≥4 weeks after the consultation. A Finnish study has demonstrated a decline in PEI scores with time elapsed since the encounter^25^ and there was some evidence of this in the current study (Supplementary Figure S1). Nevertheless, the fact that the mean time of completion post-consultation was consistent between groups supports the validity of the authors’ current findings. The impact of the COVID-19 pandemic on GP services themselves, such as the increased reliance on telephone triage systems,^26^ further complicates comparisons with the 2007 and 2016 studies.^6^^,^^7^

### Comparison with existing literature

The disparity in health needs demonstrated here are consistent with previous studies comparing deprived and affluent areas.^6^^–^8^,^^10^^,^^27^^,^^28^ Likewise, the disparity in consultation experience between deprived and affluent areas is consistent with previous studies.^6^^–^9^,^^11^^,^^29^ It is noteworthy that a previous study using the same validated measures shows remarkably similar findings to the current study (although data collection methods differed, as discussed below).^6^ This suggests that little has changed over the past decade and a half to improve the quality of GP consultations in deprived areas.

Although no previous studies have directly compared the three groups included in this study, higher patient satisfaction in RR areas compared with urban areas in general has been reported previously^30^ matched by high GP job satisfaction in RR areas.^31^ Conversely, lower patient satisfaction in DU areas has been found to be associated with lower GP job satisfaction.^9^

Differences in consultation quality between FTFC and TC have been reported previously,^32^^–^34 although not specifically in RR areas. The higher rate of TC in deprived areas is also consistent with a recent population-based study.^26^ Although higher patient satisfaction with FTFC has been shown elsewhere,^32^^–^34^,^^35^ higher enablement and perceived GP empathy scores are new findings.

### Implications for research and practice

The key implications of this study are twofold. First, the fact that FTFC were associated with better consultation experience (including perceived GP empathy) in RR areas has implications for the use of TC, since GP empathy predicts consultation outcomes^36^ and may even be associated with longer-term outcomes.^37^^,^^38^ Although this difference between FTFC and TC was not apparent in the urban groups, it should be noted that the analyses may be underpowered and further research is required to confirm or refute this.

Second, the current findings suggest the persistence of the inverse care law 4 years on from the introduction of the contract. As discussed, the authors cannot make a direct comparison with previous studies,^6^^,^^7^ so caution is required in making this assertion. However, the authors’ ongoing longitudinal analysis of the Scottish Government patient surveys from 2010 to 2023 will help support or refute this.

A key envisaged mechanism for reducing health inequalities in the 2018 contract was the provision of longer GP consultations for patients with complex multimorbidity, made possible by reducing GP workload through the expansion of the primary care MDT. However, the contract does not directly control the way practices organise care, nor does it offer financial incentives for adopting this mechanism. The extent to which the contract has enabled GPs to spend more time with patients with complex needs was not assessed in the current study, but the authors’ recent qualitative research suggests this is not happening, especially in deprived areas,^16^^,^^17^ even though evidence suggests this would be both cost-effective and beneficial to those patients with multimorbidity living in deprivation.^14^^,^^15^ This is being further explored by ongoing research on consultation length measured by routine electronic computer records, as part of the current funded programme of research.

The Scottish GP contract is an example of the global efforts to transform the delivery of primary care, with MDT expansion being a critical component, but there is a dearth of evidence reflecting patients’ experiences of these efforts.^12^ The current findings, therefore, offer potential learning for all primary care systems undergoing change. Clearly, the COVID-19 pandemic has had a major impact on the progress of the new contract in Scotland. Nevertheless, the current findings suggest that urgent steps must be taken by the Scottish Government to reverse the inverse care law and help GPs tackle the health inequalities that blight the lives of Scotland’s most vulnerable people.

## Data

The authors do not have ethical permission or patient consent to share the full data.
